# Arterial blood supply variation in the anterior midline mandible: Significance to dental implantology

**DOI:** 10.1186/s40729-015-0026-y

**Published:** 2015-09-30

**Authors:** Joseph Gakonyo, Fawzia Butt, Philip Mwachaka, Evelyn Wagaiyu

**Affiliations:** 1 Department of Conservative and Prosthetic Dentistry, University of Nairobi, P.O. Box 54954-00200, Nairobi, Kenya; 2 Department of Human Anatomy, University of Nairobi, P.O. Box 25361-00603, Nairobi, Kenya; 3 Department of Periodontology/Community and Preventive Dentistry, University of Nairobi, P.O. Box 30197, Nairobi, Kenya

**Keywords:** Sublingual artery, Dental implant, Mandibular midline foramen, Haemorrhage

## Abstract

**Background:**

Inter-foraminal implant placement poses a risk to the sublingual artery as it enters the mandibular midline lingual foramen. Lack of consensus on the source of this artery poses a dilemma to surgeons during management of haemorrhagic episodes. Determination of the exact source of this artery is therefore pivotal.

**Methods:**

This was a cross-sectional descriptive study involving 34 adult human cadavers. The facial and lingual arteries were followed from the external carotid artery to determine whether they terminated as the sublingual artery. Statistical significance tests were done using the Mann-Whitney *U* test and Pearson product-moment correlation.

**Results:**

There were 30 (88.2 %) males and 4 (11.8 %) females (male/female = 15:2) aged between 25 and 40 years. The origin of the sublingual artery was mainly from the lingual artery (73.5 %), the submental artery (17.6 %), or an anastomotic branch from the two arteries (8.9 %). The mean distance between the mandibular midline lingual foramen (MMLF) and the inferior border of the mandible was 15.58 mm (range 11.03–19.62 mm). The mean thickness of the mandible at the level of the MMLF was 10.89 mm (range 8.00–12.91 mm). No statistically significant difference was found between the two genders with regard to the morphometric measurements.

**Conclusions:**

The sublingual artery that enters the MMLF was found to be the sublingual artery as either a branch of the lingual artery (73.5 %), the submental artery (17.6 %) or an anastomosis of the lingual and submental arteries (8.9 %).

## Background

The inter-foraminal region of the human mandible is a common elective area for several dental surgery procedures due to its favourable anatomic conditions [1, 2]. Some of the procedures done in this region include insertion of endosseous dental implants, bone harvesting from the chin, genioplasty in orthognathic surgery and placement of screws during plating in management of facial fractures [3].

There have been reported cases of near fatal upper airway obstruction due to haemorrhage following implant placement in this area [4, 5]. Majority of them have been attributed to transection of the sublingual artery when a misdirected drill perforates the lingual cortex and damages the blood vessel within the surrounding soft tissues [5] as it enters the mandibular midline lingual foramen (MMLF). This vessel retracts into the floor of the mouth where a haematoma forms in the sublingual and submandibular spaces leading to a compromise of the airway, by swelling and pushing of the tongue against the palate [5, 6].

This foramen is a constant finding and has an intra-osseous part, midline mandibular lingual canal (MMLC), which contains a blood vessel that is prone to transection during routine dental implant placement [7–11]. It has been found that vessels travelling horizontal to the direction of an implant drill, but perpendicular to the spin of the drill, are at the greatest risk of laceration and transection [6, 7].

A higher risk of trauma exists in some situations like where the alveolar ridge is severely resorbed, or where trimming of the ridge has to be done either to reduce excess bone or to create a flat site for drilling, because this procedure brings the MMLC closer to the superior aspect of the mandible [11]. Drilling deeper into an extraction socket to obtain primary implant stability is now an acceptable procedure which increases the risk of bleeding. Some clinical protocols require inter-foraminal implant placement, which may lead to placement of an implant in the mandibular midline position [12].

There are two main sources of arterial blood supply to the anterior lingual area of the mandible: the submental and the lingual arteries. There is however lack of consensus as to the origin of the artery that enters the MMLF. Some authors have reported that it is a branch of the lingual artery and others the submental artery, while others have found that the vessel is an anastomosis of the two arteries [2, 6, 7, 9, 13]. The possibility of existence of anastomoses and the lack of consensus as to the source of the artery that enters the MMLF pose a dilemma of whether the submental or the lingual artery should be ligated first in the event an extra-oral approach is required to control haemorrhage [7].

There is hardly any published data on the artery that enters the MMLF. This study aimed at determining the source of the artery that enters the MMLF. It was hoped that this finding would help the surgeon to precisely ligate the feeder vessel, should there be haemorrhage during placement of an inter-foraminal dental implant.

## Methods

This was a cadaveric dissection study involving 34 human adult cadavers of indigenous Kenyan descent carried out at the Department of Human Anatomy, University of Nairobi. Majority of these cadavers are unclaimed bodies in the city of Nairobi, whose demographics are hardly known. Approval to conduct the study was obtained from the Kenyatta National Hospital/University of Nairobi Ethics, Research and Standards Committee (P694/11/2014).

The procedure involved exposure of the lingual and facial arteries, beginning from the external carotid artery and following them to determine which one of them ended as the sublingual artery. This was followed by bilateral resection of the mandible at the position of the lateral incisors.

The bucco-lingual thickness of the mandible at the level of the MMLF and the distance from the inferior border of the mandible to the MMLF were measured. All photographs were taken using a HTC digital camera (Xindian, Taiwan) with dual 4 MP, 2688 × 1520 pixels and dual-LED flash. Morphometric measurements were taken with a Pittsburgh 6-in. digital calliper (Pittsburgh, China).

Data collected were analysed using SPSS version 17 (SPSS Inc., Chicago, IL) statistical software. Probabilities that were ≤0.05 were accepted as significant. Descriptive parameters were expressed as minimum, maximum and mean ± standard deviation. The statistical significance of gender differences was determined using the Mann-Whitney *U* test.

## Results

**Fig. 1:**
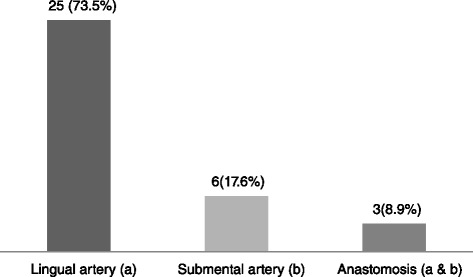
Distribution of cadavers according to source of artery entering the MMLF (*n* = 34)

**Fig. 2:**
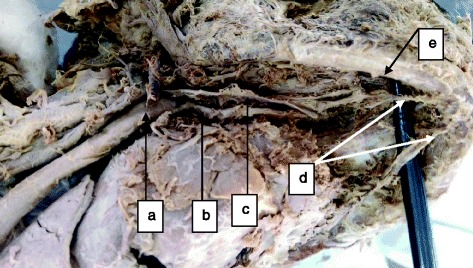
Lingual artery as the origin of the sublingual artery entering the MMLF: *a* external carotid artery, *b* lingual artery, *c* hypoglossal nerve, *d* right and left sublingual arteries originating from the ipsilateral lingual arteries and *e* inferior border of the mandible

**Fig. 3:**
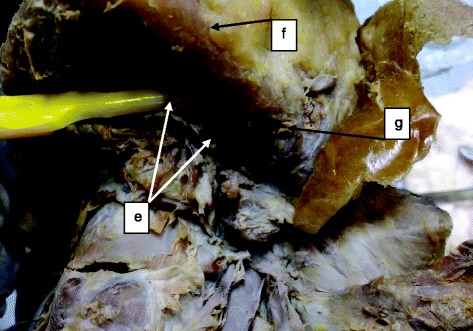
Submental artery as the source of the sublingual artery entering the MMLF: *e* submental artery, *f* inferior border of the mandible, and *g* facial artery

**Fig. 4:**
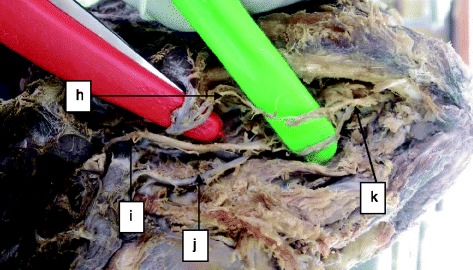
Sublingual artery originating from an anastomotic branch of the lingual and submental arteries: *h* submental artery, *i* hypoglossal artery, *j* lingual artery, and *k* anastomosis of the lingual and submental arteries

A total of 34 human cadavers were dissected in this study of which 30 (88.2 %) were males and 4 (11.8 %) were females (male/female, 15:2) aged between 25 and 40 years. The linear distance between the MMLF and the inferior border of the mandible ranged from 11.03 to 19.62 mm (mean = 15.58 mm ± 2.12 SD). The mesio-buccal thickness of the mandible at the level of the MMLF ranged between 8.00 and 12.91 mm (mean = 10.89 mm ± 1.24 SD). The source of the artery entering the MMLF came from either a branch of the lingual artery (73.5 %, *n* = 25) or submental artery (17.6 %, *n* = 6), or their anastomotic branch (8.9 %, *n* = 3) (Figs. 1, 2, 3 and 4).

A Mann-Whitney *U* test did not elicit a statistically significant difference between males and females with regard to the distance from the MMLF to the inferior border of the mandible (Table 1) or the mesio-buccal width of the mandible at the level of the MMLF (Table 2). A Pearson product-moment correlation did not elicit a statistically significant association between distance from the MMLF to the inferior border of the mandible and mesio-buccal width of the mandible at the level of the MMLF (*r* (1) = 0.062, *P* = 0.803 (two-tailed)).

**Table 1:** Distribution of distance (in mm) from MMLF to the inferior border of the mandible by gender

Gender	Number	Mean	Mann-Whitney *U* test	*P*
Male	30	17.97	46.000	0.454
Female	4	14.00

**Table 2:** Distribution of mesio-buccal width (in mm) of the mandible at the level of the MMLF by gender

Gender	Number	Mean	Mann-Whitney *U* test	*P*
Male	30	17.55	58.500	.936
Female	4	17.13		

## Discussion

The MMLF is a consistent structure through which the sublingual artery enters the mandible [8, 11]. Perforation of the lingual plate by an implant drill poses a high risk of trauma to this vessel especially when the sublingual fossa is very pronounced because this vessel courses closely to the gland and fossa [13]. When this artery is of a significant size, it is possible for 420 mL of blood to escape in 30 min leading to haematoma formation in the sublingual and submandibular spaces and leading to a compromise of the airway, by swelling and pushing of the tongue against the palate [5, 6, 14].

In a review of literature, Kalpidis et al. [7] concluded that when the haematoma is large and non-restrictive, extra-oral ligation of the feeder vessel should be applied. They did not establish the exact vessel that should be ligated to arrest bleeding. This was due to the differences in study findings on the prevalence of either the submental or the lingual artery as the main source of blood supply to the floor of the mouth. They thus suggested that in every case, the decision to ligate the facial or the lingual artery should be guided by the operator’s judgment.

In the present study, the sublingual artery appeared to originate mainly from the lingual artery (73.5 %) which was similar to the findings of Hofschneider et al. [13] (70 %, *n* = 17) and Loukas et al. [6] (73 %, *n* = 100). Our results however contradict those of previous studies that found the sublingual artery to originate from the lingual artery in 100 % of the cadavers dissected [2, 8, 9, 15]. The results of these previous studies should however be interpreted with caution because a majority of them examined one to two cadavers.

In a cadaveric study by Bavitz et al. [16], it was found that the submental artery was the main arterial blood supply to the floor of the mouth (53 %). Consequently, they suggested that the submental artery should be ligated first during extra-oral ligation to control haemorrhage in the floor of the mouth. Conversely, the sublingual artery appeared to originate from the submental artery in less than a quarter (17.6 %) of the cadavers in the present study. Our results are similar to those of Loukas et al. [6] (27 %).

The sublingual and submental arteries normally anastomose through their muscular mylohyoid branches [7]. Loukas et al. [6] only identified anastomotic patterns between sublingual and submental arteries in 40 % of the dissected cadavers, with no case where the two terminate as a single sublingual artery. We found three (8.9 %) cases of an anastomotic branch of the submental and lingual artery forming a single sublingual artery just before entering the MMLF.

The distance from the MMLF to the inferior border of the mandible in our study ranged from 11.03 to 19.62 mm (mean = 15.58 mm ± 2.12 SD) with no statistically significant difference between the two genders (*U* = 46.000, *P* = 0.454). Oettle et al. [11] reported a distance ranging from 10.83 to 12.874 mm in a dry mandible study of both black and white South Africans. In their study, cone beam computed tomography (CBCT) was used to scan the mandibles. Their study did not find a statistically significant difference according to sex or race. Soft tissue remnants on the surface of the mandibles in our study could explain the higher morphometric measurements.

A critical buccal bone thickness of 2 mm is recommended to prevent vertical resorption [17]. A minimum bone thickness of 6 mm would be required for successful placement of a standard-sized ±4-mm-diameter implant [11]. In the current study, the bucco-lingual thickness of the mandible at the level of the MMLF ranged from 8.00 to 12.91 mm (mean = 10.89 mm ± 1.24 SD). Thus, all the cadavers in our study had adequate bone thickness needed for implant placement without perforating the lingual cortical plate and traumatizing the sublingual artery.

A notable limitation in our study was a lower number of female cadavers available. Most of these cadavers are unclaimed bodies from mortuaries in Kenya. Generally, there are more male unclaimed bodies than female bodies. The Mann-Whitney *U* test was therefore used to compare the two genders.

## Conclusions

Within the limitations of this study, the sublingual artery that enters the MMLF was found to be the sublingual artery as either a branch of the lingual artery (73.5 %), the submental artery (17.6 %) or an anastomosis of the lingual and submental arteries (8.9 %). We suggest that extra-oral ligation of the lingual artery should be done first followed by ligation of the submental artery (or its parent facial artery) in case of continued bleeding. Studies with a larger sample of females should be conducted to confirm the findings of this study.
